# Impact of hypertensive disorders of pregnancy on maternal and neonatal outcomes of twin gestation: a systematic review and meta-analysis

**DOI:** 10.3389/fped.2023.1210569

**Published:** 2023-08-21

**Authors:** Xiaoqing Wu, Huifeng Gu, Junlin Wang

**Affiliations:** ^1^ Intensive Care Unit, Huzhou Maternity & Child Health Care Hospital, Huzhou, China; ^2^ Nursing Department, Huzhou Maternity & Child Health Care Hospital, Huzhou, China; ^3^ Obstetrical Department, Huzhou Maternity & Child Health Care Hospital, Huzhou, China

**Keywords:** twin pregnancy, hypertension, preeclampsia, neonatal, maternal

## Abstract

**Background:**

The impact of hypertensive disorders of pregnancy (HDP) on outcomes of twin gestations is not clear. We aimed to collate data via this meta-analysis to examine how HDP alters maternal and neonatal outcomes of twin gestations.

**Methods:**

Studies comparing pregnancy outcomes of twin gestations based on HDP and published on the databases of PubMed, CENTRAL, Scopus, Web of Science, and Embase between 1 January 2000 to 20 March 2023 were eligible for inclusion.

**Results:**

Twelve studies were included. A cumulative of 355,129 twin gestations were analyzed in the current meta-analysis. The pooled analysis found that the presence of HDP increases the risk of preterm birth (OR: 1.86 95% CI: 1.36, 2.55 *I*^2 ^= 99%) and cesarean section in twin gestations (OR: 1.36 95% CI: 1.20, 1.54 *I*^2 ^= 89%). Meta-analysis showed a significantly increased risk of low birth weight (OR: 1.30 95% CI: 1.10, 1.55 *I*^2 ^= 97%), small for gestational age (OR: 1.30 95% CI: 1.09, 1.55 *I*^2 ^= 96%) and neonatal intensive care unit admissions (OR: 1.77 95% CI: 1.43, 2.20 *I*^2 ^= 76%) with HDP in twin gestations. There was no difference in the incidence of 5-min Apgar scores <7 (OR: 1.07 95% CI: 0.87, 1.38 *I*^2 ^= 79%) but a lower risk of neonatal death (OR: 0.39 95% CI: 0.25, 0.61 *I*^2 ^= 62%) with HDP.

**Conclusion:**

HDP increases the risk of preterm birth, cesarean sections, low birth weight, SGA, and NICU admission in twin gestations. Contrastingly, the risk of neonatal death is reduced with HDP. Further studies are needed to corroborate the current results.

**Systematic Review Registration:**

PROSPERO (CRD42023407725).

## Introduction

The use of assisted reproductive technology has metamorphosed the management of infertility in recent times (1). However, as a corollary, there has been an upward trend in the incidence of twin and multiple pregnancies worldwide (2). Research has documented that twin gestations have significantly inferior outcomes as compared to singleton pregnancies (3). Mothers with twin gestation have a higher risk of gestational diabetes and hypertensive disorders of pregnancy (HDP) while their infants have an increased risk of fetal growth restriction and neonatal death (3).

HDP along with gestational hypertension and preeclampsia are among the most common causes of adverse maternal and neonatal outcomes in pregnancy. HDP is known to escalate the risk of cesarean section, preterm birth, low birth weight, small for gestational age (SGA), neonatal admission, and neonatal death in singleton pregnancies (4). Given that about 10% of pregnancies around the world are affected by HDP, the burden of adverse events is indeed huge (5). Moreover, the risk of HDP increases proportionately with multiple gestations and is as high as nearly 20% for twin gestations (6, 7). Such increased risk has been primarily associated with higher placental mass causing increased circulating levels of the anti-angiogenic molecule sFlt1 in twin gestations, which is postulated in the pathophysiology of the disease (8).

Despite the high prevalence of HDP and increasing rates of twin gestations, the impact of HDP on the outcomes of twin pregnancies is still unclear. Are the risk of adverse maternal and neonatal outcomes similar to those of singleton pregnancies or does HDP further heighten the occurrence of deleterious events? Literature comparing pregnancy outcomes of twin gestations with and without HDP is scarce and conflicting (9–11). To date, no study has comprehensively consolidated the available data to present clarity on the effects of HDP in twin pregnancies. To overcome this deficiency in literature, the present review was designed to assess the impact of HDP on maternal and neonatal outcomes of twin gestations.

## Material and methods

### Search details

The review protocol was registered on PROSPERO (CRD42023407725) and the PRISMA statement reporting guidelines were followed (12). Two reviewers conducted the literature search separately. The databases included PubMed, CENTRAL, Scopus, Web of Science, and Embase. Google Scholar was searched separately for gray literature. All articles available online between 1 January 2000 to 20 March 2023 were eligible for inclusion. There was no restriction on the language of publication.

We combined free-text and MeSH keywords with Boolean operators (AND/OR) for the literature search. The search terms included “hypertensive disorders of pregnancy”, “gestational hypertension”, “preeclampsia”, “eclampsia”, “twin”, “pregnancy”, and “gestation”. The PubMed search strategy is presented in detail in Supplementary Table S1. Similar search threads were used for all other databases.

The search results were de-duplicated and the remaining records were carefully screened based on the eligibility criteria. Non-relevant studies were excluded based on title/abstract screening. The remaining studies underwent full-text analysis for inclusion in the review. Any disagreements were solved by consensus. The references list of eligible articles was hand searched for additional articles.

### Inclusion criteria

Based on the PECOS, the inclusion criteria were: (1) Population: women with twin pregnancies, (2) Exposure: diagnosis of HDP (3) Comparison: No HDP 4: Outcomes: Any maternal and neonatal pregnancy outcomes. We quantitatively analyzed an outcome if data was reported by at least three studies.

HDP was defined as a diagnosis of either gestational hypertension, preeclampsia, or eclampsia. Gestational hypertension was defined as new-onset hypertension recorded on two occasions after 20 weeks of gestation. Preeclampsia was defined as gestational hypertension with a new onset of proteinuria, or involvement of one of the systemic organ systems. Eclampsia was defined as hypertension with proteinuria with generalized seizures or coma and could include pathologic edema. Outcomes were not pre-defined per-se and all definitions by the included studies were acceptable.

Studies comparing outcomes with singleton pregnancies, not reporting any maternal or neonatal adverse outcome, those with duplicate/overlapping data, reviews and editorials were excluded. If two or more articles used the same dataset from the same period, the study with the highest number of patients was included.

### Data management and study quality

Data on the author's last name, year of publication, location, study type, and outcomes were extracted. Also, the reviewers gathered data on the following maternal characteristics: mode of conception, HDP type, sample size, age, primiparity, smokers, obesity, gestational diabetes, and chorionicity. Two reviewers were independently involved in data collection. For maternal outcomes, sufficient data were available for pre-term birth (<37 weeks) and risk of cesarean section. For neonatal outcomes, meta-analysis was conducted for low birth weight (<2,500 g), SGA (<10th percentile), 5-min Apgar score <7, neonatal death, and neonatal intensive care unit (NICU) admission.

Two authors judged the study's quality based on Newcastle Ottawa Scale (NOS) (13). The NOS has three domains: representativeness of the study cohort, comparability, and measurement of outcomes. Points are given depending on the NOS questions. The final score of a study can range from 0 to 9.

### Statistical analysis

Statistical analysis was done using “Review Manager” [RevMan, version 5.3; Nordic Cochrane Centre (Cochrane Collaboration), Copenhagen, Denmark; 2014]. Crude dichotomous data on outcomes were sourced from studies and combined to generate an odds ratio (OR) with 95% confidence intervals (CI) in a random-effects model. The *I*^2^ statistic was the tool to determine inter-study heterogeneity. *I*^2^ < 50% meant low and >50% meant substantial heterogeneity.

## Results

**Figure 1:**
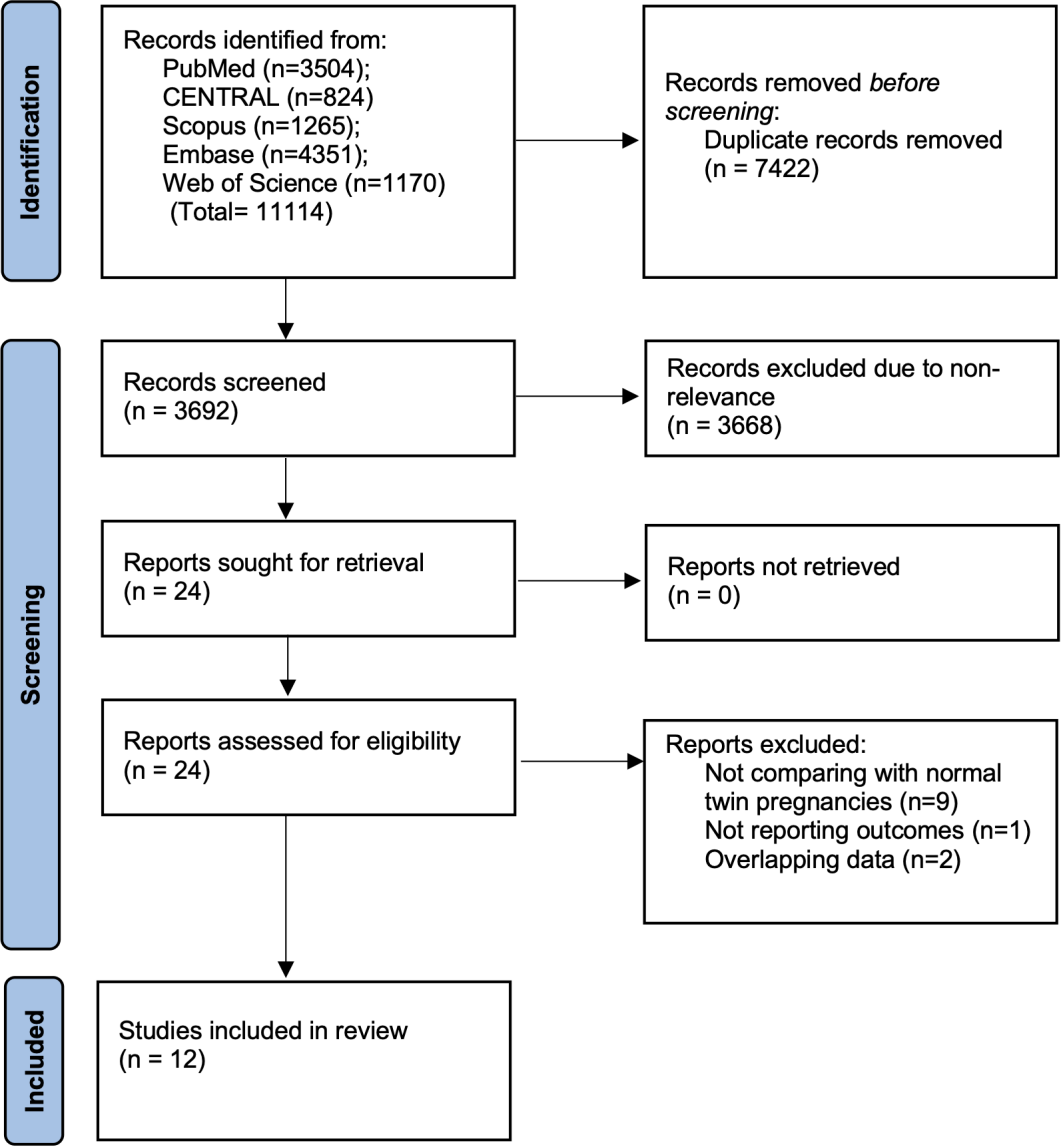
Study flowchart.

On completing the literature search and deduplication of data, a total of 3,692 articles were found (Figure 1). The reviewers examined these articles for primary eligibility and 3,668 were excluded due to non-relevance. The 24 studies which were selected for full-text analysis underwent detailed examination and 12 were found to be appropriate based on the inclusion criteria (9, 10, 21, 22, 11, 14–20). The remaining 12 studies were excluded for reasons mentioned in Figure 1.

All articles were published between 2012 and 2022. Six of the studies (9, 10, 16, 17, 20, 21) were from North America (USA or Canada) (Table 1). The remaining were from Slovenia (15), Italy (11), the UK (22), Ireland (18), and China (19). One study (9) included only assisted reproductive technology-based gestations, while all others (10, 11, 22, 14–21) included natural conception as well. Seven studies (9, 10, 16, 18, 20–22) included twin pregnancies with gestational hypertension and preeclampsia. Three studies (11, 14, 17) included only gestational hypertension and two (15, 19) included only preeclampsia. A cumulative of 355,129 twin gestations were analyzed in the included cohorts. The mean age of the females was ≤36 in all studies. The majority of studies did not report data on the prevalence of smokers, obesity, and gestational diabetes in their cohorts. Two studies (10, 21) included only dichorionic twin gestations while others included both monochorionic and dichorionic twins. The studies received a NOS score of 6 to 8.

**Table 1:** Details of included studies.

Study	Location	Conception	HDP type	Groups	Sample size	Age	Primaparous (%)	Smoker (%)	Obese (%)	Gestational diabetes (%)	Chorionicity	NOS score
Liu et al. (9)	USA	ART	GH, PE	HDP No HDP	10,817 41,903	35.6 35	38.4 33.7	0.3 0.4	15.8 12.3	NR	NR	8
Aviram et al. (20)	Canada	Mixed	GH, PE	HDP No HDP	2,120 12,556	32.7 32	60.9 41.3	6.1 8.5	26.8 17.3	14.7 10	NR	8
Hayes-Ryan et al. (14)	Ireland	Mixed	GH	HDP No HDP	270 1,296	NR	54.4 39.4	NR	24.8 74.1	8.5 7.7	Mixed	8
Giorgione et al. (22)	UK	Mixed	GH, PE	Total sample	1,473	33	NR	5.2	NR	NR	Mixed	8
Proctor et al. (21)	Canada	Mixed	GH, PE	Total sample	1,520	34.1	62.7	NR	NR	NR	Dichorionic	8
Yuan et al. (19)	China	Mixed	PE	HDP No HDP	143 367	29 29	69.9 68.9	NR	NR	NR	Mixed	8
Sparks et al. (10)	USA	Mixed	GH, PE	HDP No HDP	136 321	34.7 34.2	72.1 62	NR	NR	NR	Dichorionic	8
Hehir et al. (18)	Ireland	Mixed	GH, PE	HDP No HDP	92 885	NR	NR	NR	NR	NR	Mixed	6
Ferrazzani et al. (11)	Italy	Mixed	GH	HDP No HDP	196 912	31 31	60.1 57.3	NR	NR	NR	NR	6
Luo et al. (17)	USA	Mixed	GH	HDP No HDP	22,839 2,55,982	NR	59.6 39.7	7.5 10.8	NR	NR	NR	8
Fox et al. (16)	USA	Mixed	GH, PE	Total sample	578	34.1	61.8	NR	8.3	NR	Mixed	6
Lučovnik et al. (15)	Slovenia	Mixed	PE	HDP No HDP	181 542	30 30	NR	9.4 9.2	NR	6.6 3.1	Mixed	6

ART, assisted reproductive technology; GH, gestational hypertension; PE, Preeclampsia; NR, not reported; HDP, hypertensive disorders of pregnancy.

**Figure 2:**
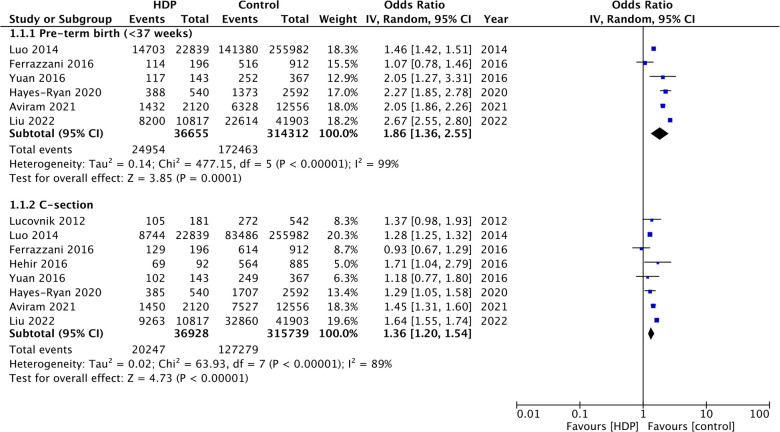
Meta-analysis of preterm birth and cesarean section with and without HDP in twin gestations.

Six studies (9, 11, 14, 17, 19, 20) reported data on preterm birth. The pooled analysis found that the presence of HDP increases the risk of preterm birth in twin gestations (OR: 1.86 95% CI: 1.36, 2.55 *I*^2 ^= 99%) (Figure 2). Similarly, the risk of cesarean section was also significantly increased with the presence of HDP in twin gestations (OR: 1.36 95% CI: 1.20, 1.54 *I*^2 ^= 89%) (Figure 2).

**Figure 3:**
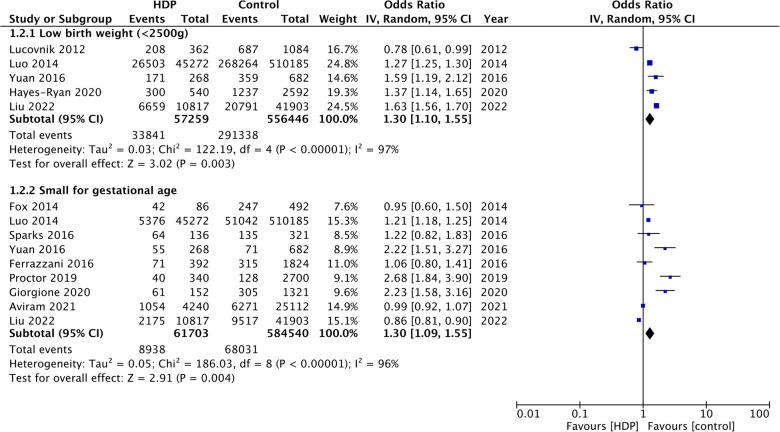
Meta-analysis of low birth weight and SGA with and without HDP in twin gestations.

**Figure 4:**
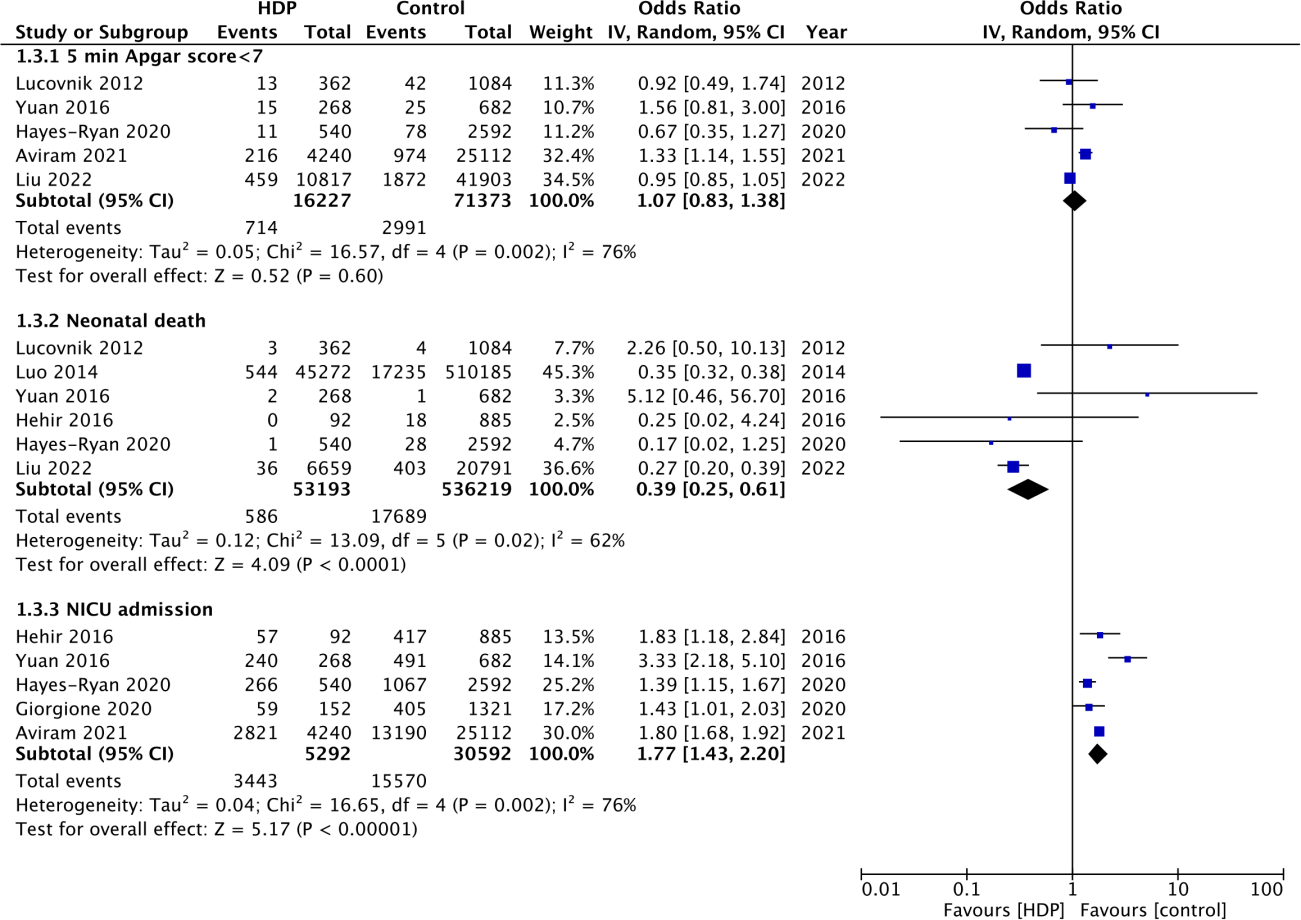
Meta-analysis of 5-min Apgar score <7, neonatal death and NICU admission with and without HDP in twin gestations.

Five studies (9, 14, 15, 17, 19) reported data on the incidence of low birth weight. Meta-analysis showed a significantly increased risk of low birth weight in twin gestations with HDP (OR: 1.30 95% CI: 1.10, 1.55 *I*^2 ^= 97%) (Figure 3). Data on the incidence of SGA was reported by nine studies (9–11, 16, 17, 19–22). On pooled analysis, a significantly high risk of SGA was seen with HDP in twin gestations (OR: 1.30 95% CI: 1.09, 1.55 *I*^2 ^= 96%) (Figure 3). Five studies (9, 14, 17, 19, 20) reported data on Apgar scores. Meta-analysis showed no difference in the incidence of 5-min Apgar scores <7 in the two groups (OR: 1.07 95% CI: 0.87, 1.38 *I*^2 ^= 79%) (Figure 4). Also, meta-analysis showed an increased risk of NICU admissions with HDP in twin gestations (OR: 1.77 95% CI: 1.43, 2.20 *I*^2 ^= 76%) but a lower risk of neonatal death (OR: 0.39 95% CI: 0.25, 0.61 *I*^2 ^= 62%) (Figure 4).

## Discussion

After combining data from 12 studies (9, 10, 21, 22, 11, 14–20), we noted that HDP has a mixed effect on pregnancy outcomes of twin pregnancies. For maternal outcomes, the presence of HDP increased the risk of preterm birth and cesarean sections. However, for neonatal outcomes, HDP reduced the risk of neonatal death while increasing the risk of low birth weight, SGA, and NICU admission.

HDP continues to be a major comorbidity affecting women during gestation. Research from several regions has confirmed the fact that the risk of HDP increases significantly in twin gestations. The CoNARTaS study from Scandinavian countries conducted between 1988 and 2007 has shown that the risk of HDP is two-fold with twin gestations and assisted reproductive technology has little impact on the incidence of HDP (23). Another study by Laine et al. (24) has found the risk of preeclampsia to be three to four times with twin gestations which is independent of maternal age, parity, educational level, smoking, comorbidities, and use of in-vitro fertilization. Similarly, another Australian study has also noted a twofold elevated risk of both gestational hypertension and preeclampsia among twins vs. singleton pregnancies (25). Despite such confirmatory research, the reason behind an increased risk of HDP remains unclear. Women with twin gestations have significantly higher weight gain compared to singleton pregnancies which in turn increases cardiac output and further increases blood pressure (26, 27). Furthermore, since the definition of HDP is the same for both singleton and twins, this directly increases the absolute incidence of HDP with twin gestations (9). Molecular experiments have shown that alteration of placental mass causing increased circulating levels of the anti-angiogenic molecule sFlt1 is an important factor behind the high risk (8). Recently, Springer et al. (28) have implicated neutrophil gelatinase-associated lipocalin as an important factor causing HDP in twin gestations. Indeed, only further research can demonstrate the pathophysiology of the increased risk of HDP in twin gestations.

The deleterious impact of HDP on maternal and neonatal outcomes is well-documented. In a recent all-encompassing meta-analysis of 152 cohort studies with 36,374,542 mothers, Li et al. (29) showed that HDP significantly increases the risk of perinatal and neonatal death, congenital malformations, fetal growth restrictions, SGA, and low birth weight. While their review also included a subgroup on twin gestations, the maximum number of studies on twin gestations was only three. Zhang et al. (30) in another review have demonstrated a significantly increased risk of congenital heart defects in neonates based on the mother's history of HDP. Bramham et al. (4) have also shown a higher risk of preterm delivery and cesarean sections in mothers with HDP. The current study, which is the first to comprehensively assess maternal and neonatal outcomes of twin gestations with and without HDP, conforms to the findings of these prior reviews. Important to note that the outcomes of our review were restricted by the data reported in the limited number of included studies and hence all relevant outcomes could not be quantitatively assessed. We found that HDP increased the risk of preterm birth and cesarean sections in mothers with twin gestations. The high rates of cesarean sections could be attributed to tendency to perform cesarean sections for mothers with preeclampsia. Studies have reported that up to 85% of women with preeclampsia deliver by cesarean section to reduce the incidence of maternal and neonatal complications (31, 32). For neonatal outcomes, HDP was found to increase the risk of low birth weight, SGA, and NICU admission but did not influence Apgar <7 scores. Recently, Wang et al. (33) have also shown that HDP increases the risk of SGA in twin gestations. However, their review was restricted to only SGA, and only seven studies were included.

An important finding of this review was the protective effect of HDP on neonatal mortality as HDP was found to reduce the risk of neonatal death by 61%. On scrutiny of the forest plot, it was noted that the outcomes were primarily influenced by two large American studies by Luo et al. (17) and Liu et al. (9). Both these administrative database studies from the USA noted a statistically significant reduction of neonatal mortality with HDP in twin pregnancies. Nevertheless, the cause of such an effect is still unknown. Luo et al. (17) speculated that antihypertensive medications may benefit fetal survival as drugs like labetalol may promote fetal lung maturation and lower mortality. Another possibility put forward was that the other unreported and serious maternal comorbidities or fetal complications could have been higher in the non-HDP group which increased neonatal mortality. Lastly, it is plausible that these results could be due to selection bias. Women with HDP could have received better ante-natal care in anticipation of complications which could have affected neonatal mortality rates. Nevertheless, further prospective studies from other countries are needed to clarify if the protective effect is real or a statistical artifact due to unmeasured confounding.

Our review was unable to examine the role of chorionicity as a confounder influencing outcomes of twin gestations affected by HDP. While a few studies focused only on dichorionic gestations the others included a mixed population of monochorionic and dichorionic pregnancies. The scarce data prevented a subgroup or a meta-regression analysis. However, Che et al. (34) have shown that outcomes of twin gestations complicated by HDP differ based on chorionicity. In dichorionic pregnancies, adverse pregnancy outcomes increase with higher grades of HDP while no such effect was noted in monochorionic twins.

There are a few limitations to our review. Firstly, the outcomes were derived from retrospective data and a small number of studies. Retrospective data is prone to bias which could affect the outcome of the systematic review. However, it is important to note that randomized controlled trials are not possible as the exposure (HDP) is a medical complication which cannot be induced. Also, the number of studies in each meta-analysis was <10. Inaccuracies in data entry could have altered the outcomes. Secondly, the two (9, 17) database studies from the USA were shown to influence outcomes due to their significantly huge sample size. Thirdly, the significant heterogeneity in the analysis is also a cause of concern. Variations in study populations, method of conception, comorbidities, obesity, gestational diabetes, chorionicity, etc. could all have influenced the outcomes and increased inter-study heterogeneity. Also, only crude outcome data were pooled in the review due to a lack of reporting of adjusted data amongst the included studies. Fourthly, studies did not report data on anti-hypertensive treatments which made it unfeasible to assess how these drugs affected pregnancy outcomes. Lastly, the scarce data made it impossible to assess the impact of assisted reproductive technology and chorionicity on outcomes.

## Conclusions

Available evidence suggests that HDP increases the risk of preterm birth, cesarean sections, low birth weight, SGA, and NICU admission in twin gestations. Contrastingly, the risk of neonatal death is reduced with HDP. Further studies are needed to corroborate the current results.

## Data Availability

Publicly available datasets were analyzed in this study. This data can be found here: The original contributions presented in the study are included in the article/**Supplementary Material**, further inquiries can be directed to the corresponding author.
