# Supplementary material for: Impact of hypertensive disorders of pregnancy on maternal and neonatal outcomes of twin gestation: a systematic review and meta-analysis
Source: Front Pediatr. 2023 Aug 21;11:1210569. doi: 10.3389/fped.2023.1210569 (PMC10475597; doi:10.3389/fped.2023.1210569)
Supplement: Supplementary file 1 [file Table1.docx]

Supplementary Table 1: Search Strategy

| **Query** | **Search Details** |
| --- | --- |
| ((((hypertensive disorders of pregnancy) OR (gestational hypertension)) OR (preeclampsia)) AND (twin)) AND (pregnancy) | ((("hypertense"[All Fields] OR "hypertension"[MeSH Terms] OR "hypertension"[All Fields] OR "hypertension s"[All Fields] OR "hypertensions"[All Fields] OR "hypertensive"[All Fields] OR "hypertensive s"[All Fields] OR "hypertensives"[All Fields]) AND ("disease"[MeSH Terms] OR "disease"[All Fields] OR "disorder"[All Fields] OR "disorders"[All Fields] OR "disorder s"[All Fields] OR "disordes"[All Fields]) AND ("pregnancy"[MeSH Terms] OR "pregnancy"[All Fields] OR "pregnancies"[All Fields] OR "pregnancy s"[All Fields])) OR ("hypertension, pregnancy induced"[MeSH Terms] OR ("hypertension"[All Fields] AND "pregnancy induced"[All Fields]) OR "pregnancy-induced hypertension"[All Fields] OR ("gestational"[All Fields] AND "hypertension"[All Fields]) OR "gestational hypertension"[All Fields]) OR ("pre eclampsia"[MeSH Terms] OR "pre eclampsia"[All Fields] OR "preeclampsia"[All Fields])) AND ("twins"[MeSH Terms] OR "twins"[All Fields] OR "twin"[All Fields]) AND ("pregnancy"[MeSH Terms] OR "pregnancy"[All Fields] OR "pregnancies"[All Fields] OR "pregnancy s"[All Fields]) |
| ((((hypertensive disorders of pregnancy) OR (gestational hypertension)) OR (eclampsia)) AND (twin)) AND (gestation) | ((("hypertense"[All Fields] OR "hypertension"[MeSH Terms] OR "hypertension"[All Fields] OR "hypertension s"[All Fields] OR "hypertensions"[All Fields] OR "hypertensive"[All Fields] OR "hypertensive s"[All Fields] OR "hypertensives"[All Fields]) AND ("disease"[MeSH Terms] OR "disease"[All Fields] OR "disorder"[All Fields] OR "disorders"[All Fields] OR "disorder s"[All Fields] OR "disordes"[All Fields]) AND ("pregnancy"[MeSH Terms] OR "pregnancy"[All Fields] OR "pregnancies"[All Fields] OR "pregnancy s"[All Fields])) OR ("hypertension, pregnancy induced"[MeSH Terms] OR ("hypertension"[All Fields] AND "pregnancy induced"[All Fields]) OR "pregnancy-induced hypertension"[All Fields] OR ("gestational"[All Fields] AND "hypertension"[All Fields]) OR "gestational hypertension"[All Fields]) OR ("eclampsia"[MeSH Terms] OR "eclampsia"[All Fields] OR "eclampsia"[All Fields])) AND ("twins"[MeSH Terms] OR "twins"[All Fields] OR "twin"[All Fields]) AND ("gestate"[All Fields] OR "gestated"[All Fields] OR "gestates"[All Fields] OR "gestating"[All Fields] OR "gestational"[All Fields] OR "gestations"[All Fields] OR "pregnancy"[MeSH Terms] OR "pregnancy"[All Fields] OR "gestation"[All Fields]) |
